# A new model for parent-of-origin effect analyses applied to Brown Swiss cattle slaughterhouse data

**DOI:** 10.1017/S1751731116002391

**Published:** 2016-12-06

**Authors:** I. Blunk, M. Mayer, H. Hamann, N. Reinsch

**Affiliations:** 1 Leibniz-Institut für Nutztierbiologie (FBN), Institut für Genetik und Biometrie, Wilhelm-Stahl-Allee 2, 18196 Dummerstorf, Germany; 2 Landesamt für Geoinformation und Landentwicklung Baden-Württemberg, Dienststelle Kornwestheim, Stuttgarter Straße 161, 70806 Kornwestheim, Germany

**Keywords:** beef trait, Brown Swiss cattle, epigenetics, imprinting variance, parent-of-origin effects

## Abstract

Genomic imprinting is a phenomenon that arises when the expression of genes depends on the parental origin of alleles. Epigenetic mechanisms may induce the full or partial suppression of maternal or paternal alleles, thereby leading to different types of imprinting. However, imprinting effects have received little consideration in animal breeding programmes, although their relevance to some agricultural important traits has been demonstrated. A recently proposed model (imprinting model) with two path-of-transmission (male and female)-specific breeding values for each animal accounts for all types of imprinting simultaneously (paternal, maternal, full and partial). Imprinting effects (or more generally: parent-of-origin effects (POE)) are determined by taking the difference between the two genetic effects in each animal. However, the computation of their prediction error variance (PEV) is laborious; thus, we propose a new model that is equivalent to the aforementioned imprinting model, which facilitates the direct estimation of imprinting effects instead of taking the differences and the PEV is readily obtained. We applied the new model to slaughterhouse data for Brown Swiss cattle, among which imprinting has never been investigated previously. Data were available for up to 173 051 fattening bulls, where the pedigrees contained up to 428 710 animals representing the entire Brown Swiss population of Austria and Germany. The traits analysed comprised the net BW gain, fat score, EUROP class and killing out percentage. The analysis demonstrated that the net BW gain, fat score and EUROP class were influenced significantly by POE. After estimating the POE, the new model yielded estimates with reliabilities ranging between 0.4 and 0.9. On average, the imprinting variances accounted for 9.6% of the total genetic variance, where the maternal gamete was the main contributor. Moreover, our results agreed well with those obtained using linear models when the EUROP class and fat score were treated as categorical traits by applying a GLMM with a logit link function.

## Implications

Genomic imprinting is an epigenetic phenomenon where the expression of genes depends on the parental origin of their alleles. Imprinting is known to affect a variety of value-determining traits in agricultural species, and thus it should be considered in animal breeding. However, the existing methods are still difficult in practice using standard statistical software. Therefore, we propose a new statistical model that allows the direct estimation of imprinting effects and their prediction error variances (PEVs). We applied this model to slaughterhouse data for Brown Swiss cattle, a breed in which imprinting has never been investigated previously.

## Introduction

Genomic imprinting is known to be caused by allele-specific DNA methylation and histone modifications during gametogenesis, which depend on the sex of an animal (for a review, see Reik and Walter, 2001). Thus, imprinting is an epigenetic phenomenon that alters the expression of genes according to the parental origin of their alleles. Therefore, imprinting effects often are referred to as parent-of-origin effects (POE), which are, however, not synonymous, as the latter include parent-of-origin-dependent effects which do not, by definition, constitute imprinting effects (e.g. maternal genetic effects as emphasised by Hager *et al*., 2008). A well-known example for genomic imprinting is the callipyge mutation in sheep, which causes extreme muscle hypertrophy that only becomes evident when the offspring inherit the mutation from their sire (Cockett *et al*., 1999). A scenario where the paternal allele of an imprinted locus is fully inactivated but the maternal allele exhibits active expression is referred to as complete paternal imprinting. The opposite scenario is defined as complete maternal imprinting. An incomplete lack of allele expression known as partial imprinting is caused by unstable imprinting patterns over time or between tissues (e.g. Gould and Pfeifer, 1998).

A series of mapping experiments based on quantitative trait loci (QTL) led to the identification of a polymorphism that causes a paternally expressed QTL in the *IGF2* region of the pig (e.g. Nezer *et al*., 1999; Van Laere *et al*., 2003). This polymorphism explained 15% to 30% of the phenotypic variation in muscle mass (Van Laere *et al*., 2003). More recently, Lopes Pinto *et al*. (2014) performed an expression study and detected parental single nucleotide polymorphisms at up to 650 loci in three different chicken tissues that indicated predominant paternal imprinting.

The first study of this type was reported by de Vries *et al*. (1994) and the analysis of variance components is now a sui generis approach in livestock genetics for investigating the importance of imprinting effects for genetic variation. The first versions of this approach used an animal model with an additional random parental effect (e.g. Engellandt and Tier, 2002) to account for either full paternal or full maternal imprinting. In 2010, Neugebauer *et al*. (2010a and 2010b) introduced a model with two additive effects (one ‘as sire’ and one ‘as dam’) per animal to account for all variants of imprinting: paternal, maternal, full and partial. Based on analyses of slaughter data, they found 19 traits in pigs (Large White) and 10 traits in cattle (German Simmental) with significant influences of POE. The imprinting variance accounted for 5% to 19% and 8% to 25% of the total genetic variance, respectively. Recently, equivalent gametic models were applied by Tier and Meyer (2012) to analyse ultrasonic measures of body composition in cattle, thereby determining an average relative imprinting variance of 28%.

To consider genomic imprinting in animal breeding programmes, Nishio and Satoh (2014) proposed a new genomic BLUP model, where they used a genomic imprinting relationship matrix constructed from paternal and maternal marker alleles to indicate an improvement in the genetic prediction reliability in a simulation study.

Nevertheless, imprinting effects are still not considered routinely during genetic evaluation. To promote the integration process, further investigations are necessary to determine the effects of imprinting on important agricultural traits. However, none of the previously proposed models used for analysing the variance components can estimate these imprinting effects directly. In addition, the computation of their PEV requires laborious procedures to evaluate their reliability (Neugebauer *et al*., 2010a and 2010b).

Therefore, in this study, we propose a new model that is equivalent to the model of Neugebauer *et al*. (2010a and 2010b), in which the direct estimation of imprinting effects is facilitated, and their PEVs can be obtained easily using existing software. To demonstrate its practical use, we applied this model to Brown Swiss cattle slaughter data. Four value-determining slaughter traits were available for Austrian and German fattening bulls. Two traits were analysed a second time by applying a generalised linear mixed model (GLMM) with a logit link function.

## Material and methods

### Beef trait data

A data set comprising 247 883 Brown Swiss fattening bulls slaughtered between 1994 and 2013 was provided by the genetic evaluation centre of the Landesamt für Geoinformation und Landentwicklung in Baden-Württemberg, Germany. This is a known dairy breed, but Brown Swiss bulls are fattened up to an end weight of ~600 kg. Data from Austria and Germany were used at regular intervals to predict breeding values for Brown Swiss and German Simmental within a joint genetic evaluation procedure for both breeds. The sires are evaluated using their progeny performance, which is routinely recorded at slaughterhouses. Thus, we used the net BW gain (carcass weight divided by age (g/days)), carcass conformation, carcass fatness and killing out percentage (carcass weight divided by life weight (%)).

The carcass conformation was defined according to the European muscle conformation system EUROP (E=excellent to P=poor). However, these EUROP grades were replaced by five monetary values (670, 655, 635, 585, 525), which reflect the fact that although prices differ over time, the price differences between classes remain stable (Engellandt *et al*., 1999a). The majority of the fattening bulls were categorised into classes O (19.54%) and R (78.45%).

Carcass fatness was available as scores ranging from 1 (lean) to 5 (very fat) where most of the animals were classified with scores of 2 (23.27%) and 3 (71.9%).

All of the fattening bulls with missing sires and/or dams, as well as all bulls belonging to a comparison group with less than five animals per group (four animals per group for the killing out percentage) were eliminated from the data set, which led to varying number of observations. The highest number was available for net BW gain (173 051) and the smallest for the killing out percentage (3226). A summary of the data is given in Table 1.

**Table 1:** Descriptive statistics, number of fattening bulls (*n*), pedigree sizes and heritabilities for the traits analysed in this study

Traits	Mean	SD	*n*	Pedigree	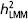	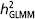
Net BW gain (g/days)	646.72	74.24	173 051	428 710	0.26 (0.01)	–
Fat score	2.81	0.50	133 671	420 626	0.22 (0.01)	0.46 (0.02)
Conformation class	625.20	20.98	133 671	420 626	0.15 (0.01)	0.43 (0.02)
Killing out percentage (%)	56.63	1.17	3226	24 329	0.52 (0.08)	–

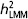
=heritability estimated using a linear animal model; 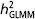
=heritability estimated using a generalised linear animal model. Standard errors are given in brackets.

The number of pedigrees was 428 710 for net BW gain (up to 21 generations), 420 626 for carcass conformation class and fat score (up to 21 generations) and 24 329 for the killing out percentage (up to 20 generations). The pedigree for net BW gain was pruned using the SECATEURS program (Meyer, 2003). This procedure considerably reduced the number of animals due to the elimination of uninformative parents for the estimation of genetic parameters.

As suggested by Westell and Van Vleck (1987), unknown parents (phantom parents) were assigned to genetic groups based on their expected year of birth. The birth years of unidentified animals were assigned according to the average generation intervals, which were estimated for four paths of selection using the software package pedig (Boichard, 2002). Those groups represented the average genetic merit of animals selected as parents on a contemporary basis. All of the phantoms that were likely to have been born before 1974 were assigned to the first group. All other groups were specified according to 3-year periods until 1996. Missing animals likely to have been born after 1996 were assigned to the last group. Furthermore, two parallel sets of groups were specified to characterise phantom sires and dams because the male and female paths of selection are assumed to differ in terms of genetic merit (Westell and Van Vleck, 1987). Thus, 18 genetic groups in total were assigned to unknown animals in each pedigree as groups based on a combination of time and sex.

### Models for analysis

#### Imprinting model

To investigate the role of imprinting effects, Neugebauer *et al*. (2010a and 2010b) developed a model with two additive genetic effects per animal, which are only estimated for the parents. This model accounts for all variants of genomic imprinting and it is known as the *imprinting model*. In matrix notation, the model is
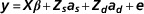
where ***y*** is a vector of observations; ***β*** a vector of fixed effects; ***a***_***s***_ (***a***_***d***_) a vector of random genetic effects under a paternal (maternal) expression pattern, which corresponds to the vector of the transmitting ability (TA) for the sire (dam); ***X***, ***Z***_***s***_ and ***Z***_***d***_ are the corresponding incidence matrices; and ***e*** a vector of random residuals. In terms of gametic variances, the variance–covariance components of random effects can be written as
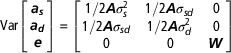



The mixed model equations are
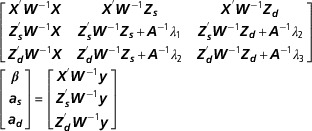
where the matrix of *λ* coefficients is equivalent to
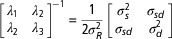



***A*** is the numerator relationship matrix and the diagonal matrix ***W*** has the elements
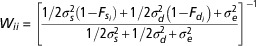
and it corrects the error variance of each observation due to the Mendelian sampling component with regard to the respective inbreeding coefficient (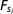, 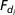) of the parents. The difference between both parental genetic effects is referred to as the imprinting effect (***i***=***a***_***s***_−***a***_***d***_) and its variance defines the imprinting variance 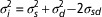. The total additive genetic variance is given by 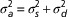, which comprises the imprinted (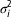) and Mendelian (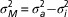) parts of inheritance.

#### The equivalent model

As mentioned earlier, the imprinting effect *i* can be derived easily as the difference between both parental TAs using the *imprinting model*. However, determining their PEVs is demanding because the off-diagonal elements of the inverted coefficient matrices of the mixed model equations are necessary (Neugebauer *et al*., 2010a and 2010b). Therefore, we propose an equivalent imprinting model that allows the direct estimation of imprinting effects as well as their PEVs. As stated by Henderson (1985), alternative models can generate a class of variance–covariance estimates that are identical to those generated by the original model after linear transformation. Our new model is equivalent to the *imprinting model* according to Henderson (1985), so we refer to it as the *equivalent model*. This model can be written as
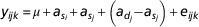
where *y*_*ijk*_ is the observation of the *k*th progeny of sire *i* and dam *j* and *μ* the overall mean. The effect 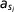 corresponds to the TA of sire *i* as sire and 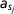 the TA of dam *j* as sire. However, the dam’s influence comprises her TA as dam, so her imprinting effect (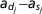) needs to be added. Thus, the dam’s TA corresponds to her TA as sire plus her imprinting effect. The effect *e*_*ijk*_ is the random residual. In terms of gametic variances, the corresponding variance–covariance components are
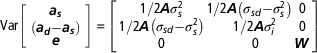
where 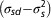 is the covariance between the TA as sire and the imprinting effect. To satisfy Henderson’s condition of equivalence, the variance–covariance components estimated using the *equivalent model* can be converted to a linear manner into those estimated using the *imprinting model*. The mixed model equations of the *equivalent model* can be written as
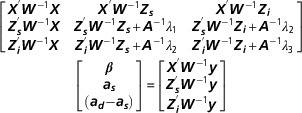



In contrast to the *imprinting model*, where the incidence matrix ***Z***_***s***_ comprises only one non-zero element per row, each row of ***Z***_***s***_ in the *equivalent model* contains two ones. The first links observations to the TA of sires as sires. The second connects observations to the TA of dams as sires. Incidence matrix ***Z***_***i***_ is identical to incidence matrix ***Z***_***d***_ in the *imprinting model* but in this case, it links observations to imprinting effects. All of the other quantities are the same as those defined earlier and the *λ* values correspond to
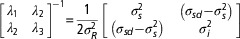



In this study, we used the estimated variance–covariance components of the *imprinting model* to predict the imprinting effects and their PEVs using the *equivalent model* in a single iteration. The PEV was then used to calculate the reliability (*r* ^2^) of the imprinting effects as follows:
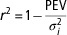



### Effects in the model

The model includes the following effects:
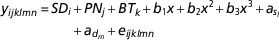
where *y*_*ijklmn*_ is a beef trait record; *SD*_*i*_ the fixed effect of the *i*th comparison group (combination of fattening farm and date of slaughter); *PN*_*j*_ the fixed effect of the *j*th parity number (first, second and more calvings); *BT*_*k*_ the fixed effect of the *k*th birth type (singleton or twin); *b* the linear (*b*_1_), quadratic (*b*_2_) and cubic (*b*_3_) regression on slaughter age *x*; 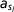 the random additive genetic effect as sire *l*; 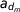 the random additive genetic effect as dam *m*; and *e*_*ijklmn*_ the random residual. It should be noted that carcass fatness is used as a fixed effect in the routine genetic evaluation. However, we treated it as a trait because it is known to be genetically influenced. Modified equations for an animal model were used to consider missing parents within genetic groups, as described by Quaas and Pollak (1981) and Westell *et al*. (1988). Y-chromosomal and mitochondrial effects were not considered because neither were found to be of significant importance for beef traits (Reinsch *et al*., 1999; Neugebauer *et al*., 2010a and 2010b). All of the variance–covariance components were estimated via the ASReml-package version 3.0 (Gilmour *et al*., 2009).

Most fattening bulls were assigned to conformation classes O and R (98%) and fat scores 2 and 3 (95%), so both traits were also treated as ordered categorical traits with binomial distributions. Thus, fattening bulls were classified either to class zero (conformation classes E, U and R; fat scores 1 and 2) or to class one (conformation classes O and P; fat scores 3, 4 and 5). A logit link was chosen for the GLMM because there is an anecdotal evidence that logit GLMM converges better than probit GLMM when the variance components are estimated using the pseudo-likelihood approach of Gilmour *et al*. (2009). The probability that an observation with index *k* belongs to class zero is
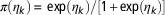
where the linear predictor is
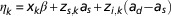
and *x*_*k*_, *z*_*s*,*k*_ and *z*_*i*,*k*_ are the *k*th rows of the aforementioned incidence matrices ***X***, ***Z***_***s***_ and ***Z***_***i***_, respectively. The vectors ***β***, ***a***_***s***_ and (***a***_***d***_−***a***_***s***_) are defined as described in the corresponding linear models.

### Test of hypotheses

Tests for significant imprinting variance were performed as described by Neugebauer *et al*. (2010a and 2010b). The null hypothesis assumed no imprinting effects, whereas the alternative hypothesis implied their existence. Two models were fitted per trait. The first corresponds to the *imprinting model* and the second to an animal model. We determined the model with the best fit to the data, thereby testing for the existence of significant imprinting effects, by comparing the REML log-likelihoods of both models using a REML likelihood ratio test (RLRT). The RLRT is asymptotically distributed as a mixture of two *χ* ^2^ distributions with 1 and 2 DF (Self and Liang, 1987). The mixture proportions deviate from 1 : 1 with correlated observations and they are difficult to determine, so we applied a conservative test with a *χ* ^2^ distribution with 2 DF (Neugebauer *et al*., 2010a and 2010b). This testing technique is only valid for linear mixed models (LMM). Using the GLMM, the ASReml-package employs an approximate likelihood (penalised quasi-likelihood) that cannot be used to test differences (Gilmour *et al*., 2009).

## Results and discussion

### The equivalent model

According to Henderson’s (1985) condition of equivalence, the *imprinting model* and *equivalent model* were assumed to yield the same results after linear transformation. The satisfaction of this condition was formally proved (Supplementary Material S1) and verified using simulated data sets from a previous study (Blunk and Reinsch, 2014). Moreover, we applied the *equivalent model* to Brown Swiss data in case the imprinting variance was significant. The *equivalent model* was found to require more iterations to converge in likelihood, which may differ when the variance components change. However, given the corresponding variance components, the major advantage of this method is that the desired effects and their PEVs can be achieved within a single iteration using software packages such as ASReml. Further computations are not necessary because only the diagonal elements of the inverted coefficient matrix are required. For the *equivalent model* as a GLMM, a single iteration may not be sufficient because logit analyses are performed on an underlying scale using a working variable, which takes several iterations to stabilise. After the variable stabilised, the genetic parameters estimated using the *equivalent model* as a GLMM agreed completely with the genetic parameters estimated using the *imprinting model* as a GLMM.

In large routine applications inverting the coefficient matrix and the exact calculation of PEVs may become infeasible for either model. Consequently, approximations would be useful in such situations, as they already have been developed for different kinds of models (see e.g. Tier and Meyer, 2004 and the references herein). Such approximations, however, need to be evaluated if they work satisfactorily for the data structure of a certain breed or trait and the *equivalent model* may be a useful tool for that purpose.

It should be noted that the *equivalent model* relates three genetic effects to each observation: the TA of sire *i* as dam (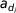) and dam *j* as dam (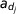) plus the imprinting effect 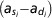. Alternatively, the imprinting effect could be defined with an opposite sign as 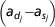, which clearly leaves the imprinting variance unaffected. Then the three genetic effects were as follows: the TA of sire *i* as sire (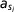) and dam *j* as sire (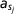) plus the imprinting effect 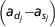. The covariance between the imprinting effect and the two possible types of TA in the model is either 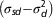 or 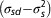. Both covariances represent negative parental contributions to the imprinting variance (Neugebauer *et al*., 2010a and 2010b) and, when signs are reversed, add up to 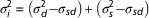. Hence, their sum must be positive although a single covariance may become negative.

### Reliability of parent-of-origin effects and genetic trends

The ease of PEV computation using the *equivalent model* facilitated a closer inspection of the reliability of the predicted POE despite the huge number of animals included. For the net BW gain, the reliability of the POE ranged from 0.38 to 0.89 for sires and from 0.38 to 0.94 for dams, with an average of 0.56 for both sexes. The reliability of the POE on the fat scores ranged from 0.38 to 0.91 for sires and 0.38 to 0.70 for dams, with an average of 0.54 for both sexes. For the conformation class, the reliability of the POE ranged from 0.38 to 0.89 for sires and 0.38 to 0.68 for dams, with an average of 0.54 for both sexes. The reliability of the POE generated using the GLMM had a slightly wider range from 0.37 to 0.93 for both traits.

In general, the reliability of genetic estimates depends mainly on the availability of data such as individual records and kinship information (Mrode, 2014). The amount of kinship information depends mainly on the number of progeny. In the present study, a high number of male progeny was needed per animal, but a high number of daughters and maternal grandsons was also a necessary prerequisite because the *imprinting model* includes the genetic effect as sire as well as the genetic effect as dam. This was highlighted by our analysis of given family structures. For example, sires with differences in the average reliability for the estimated POE on the net BW gain had different average numbers of sons, daughters and maternal grandsons as follows.

**Table:** 

*r* ^2^<0.55	4.54	10.81	5.27
0.55⩽*r* ^2^⩽0.65	10.90	15.79	8.417
*r* ^2^>0.65	55.88	484.64	106.31

**Figure 1:**
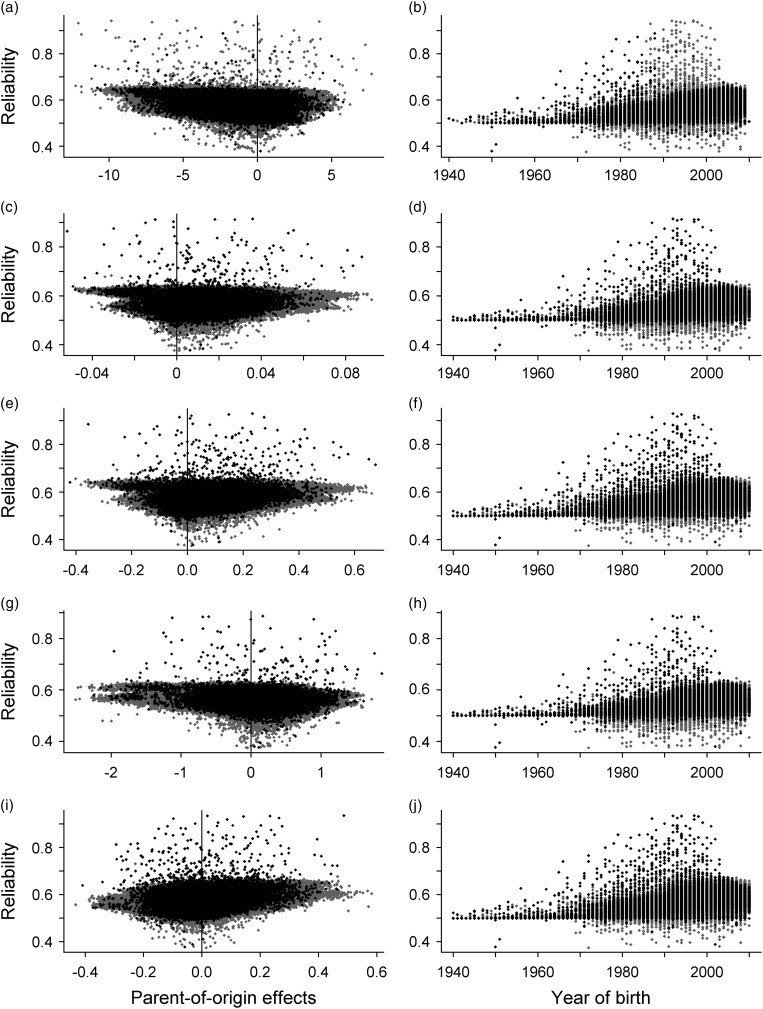
Parent-of-origin effects for sires (black) and dams (grey) relative to their reliability (left side), as well as their reliability relative to the year of birth (right side). Parent-of-origin effects were estimated using a linear mixed model for the net BW gain (g/days) (a, b), fat score (c, d) and conformation class (g, h). Parent-of-origin effects were estimated using a generalised linear mixed model for the fat score (e, f) and conformation class (i, j).

The left-hand side of Figure 1 shows the POE estimated for each individual (horizontal axes) using the *equivalent model* as LMM and GLMM relative to its reliability. The regression of less reliable POE to their expected mean of 0 was observed for all traits. By contrast, more reliable effects exhibited increasing variation, most of which could be assigned to male animals because males are biologically capable of having more kinship information than females. An exception was the net BW gain, where the most reliable POE could be assigned to females. These animals were mostly bull dams with sons, which were also sires of many sons and daughters. This yielded an informative family structure, which was facilitated by the large amount of pedigree available for net BW gain.

The panels on the right in Figure 1 illustrate the changes in the reliability of the POE for animals born from 1940 to 2010. An increase, especially for males, was followed by a drop in the presence of the top reliabilities from about 2000. This increase was related to the growing amount of available data collected from 1994. However, younger animals had less opportunity to accumulate information from grandsons, which explains the lack of top reliabilities (>0.65) in the more recent birth cohorts.

**Figure 2:**
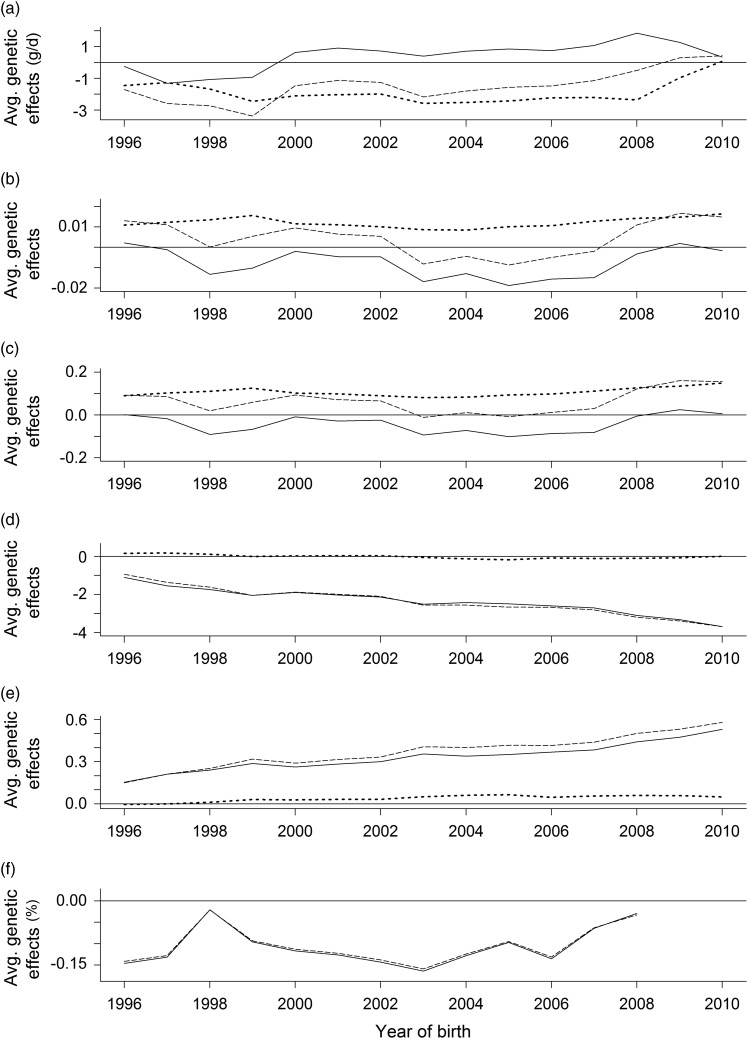
Average parent-of-origin effects (dotted line) and transmitting abilities for animals as sire (solid line) and as dam (dashed line) relative to the year of birth. The genetic effects were estimated using a linear mixed model for the net BW gain (a), fat score (b), conformation class (d) and killing out percentage (f). A generalised linear mixed model was used for the fat score (c) and conformation class (e).

Overall, the genetic trends in the TA and POE appeared to be fairly constant, with the exception of a clear undesired trend in the conformation class, which was almost identical in the LMM and GLMM (Figure 2). This trend is attributable to a correlated response to intense selection for milk performance in Brown Swiss, as well as the slightly positive genetic trend in the net BW gain.

### Significance of imprinting variances

Significant imprinting variances were found for the net BW gain, fat score and conformation class (Table 2), where the error probabilities were all <0.001. The estimated imprinting variance accounted for 10.58% of the total additive genetic variance in the net BW gain, as well as 9.17% for the fat score and 9.12% for the conformation class. The imprinting variances were driven by both deviating parental variances and imperfect correlations of about 0.9 between parental effects. It should be mentioned that this correlation was not constrained to one (Tier and Meyer, 2012) to ensure that the test for the existence of the imprinting variance remained as general as possible.

**Table 2:** Genetic parameters, correlation coefficient, test statistic and variance component ratios estimated using linear and generalised linear mixed models with two additive genetic effects per animal for all traits

Traits	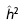	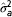	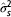	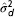	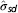	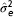	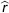	RLRT^1^	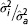	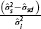	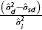
Net BW gain (g/days)	0.28 (0.01)	804.960 (34.870)	359.660 (18.48)	445.300 (25.468)	359.890 (18.074)	2071.200 (27.362)	0.899 (0.020)	122.8***	10.58 (2.10)	−0.3 (16.8)	100.3 (16.8)
Fat score	0.23 (0.01)	0.044 (0.002)	0.021 (0.001)	0.024 (0.002)	0.020 (0.001)	0.149 (0.002)	0.910 (0.022)	69.2***	9.17 (2.20)	12.3 (22.2)	87.7 (22.2)
Fat score^2^	0.46 (0.02)	1.960 (0.085)	0.790 (0.052)	1.170 (0.061)	0.869 (0.048)	3.290	0.904 (0.025)	–	11.31 (2.58)	−35.7 (20.7)	135.7 (20.7)
Conformation class	0.16 (0.01)	58.614 (3.900)	27.027 (1.980)	31.587 (2.850)	26.634 (1.980)	320.080 (3.289)	0.912 (0.029)	27.6***	9.12 (2.80)	7.3 (28.3)	92.7 (28.3)
Conformation class^2^	0.43 (0.02)	1.796 (0.083)	0.678 (0.049)	1.119 (0.062)	0.773 (0.048)	3.290	0.888 (0.031)	–	13.95 (3.26)	−37.9 (20.0)	137.9 (20.0)
Killing out percentage (%)	0.52 (0.09)	1.348 (0.267)	0.701 (0.138)	0.648 (0.201)	0.672 (0.135)	1.262 (0.221)	0.998 (0.096)	0.7	–	–	–

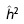
=heritability; 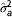
=additive genetic variance; 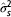
=additive genetic variance as sire; 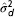
=additive genetic variance as dam; 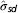
=covariance; 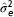
=residual variance; 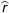
=correlation between parental effects; 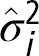
=imprinting variance; 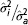
=relative imprinting variance (%); 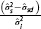
=paternal contribution to the imprinting variance (%); 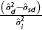
=maternal contribution to the imprinting variance (%). Standard errors are given in brackets.

1 REML likelihood ratio test; RLRT=2 (log-likelihood_imprinting model_−log-likelihood_animal model_).

2 Treated as ordered categorical traits using a generalised linear mixed model.

***P<0.001.

The significances could not be tested formally, but the imprinting variances obtained from the GLMM accounted for slightly higher proportions of the total genetic variance. The ratios of 11.31% and 13.95% were in good agreement with the proportions obtained from the LMM (Table 2), although the absolute values are not directly comparable because the GLMM operates on an unobservable logit scale (Dempster and Lerner, 1950).

The analysis yielded no significant results for the killing out percentage (*P*=0.704). The incorporation of genetic groups had no noticeable impact on the estimates of genetic parameters for all of the traits analysed.

There are no comparable studies with respect to POE in Brown Swiss cattle, so comparisons were made with the POE analysis conducted in German Simmental by Neugebauer *et al*. (2010b). In contrast to our findings, they found that the killing out percentage was significantly affected by POE, where the imprinting variance accounted for 24% of the total genetic variance. However, we only had 3226 observations, so our study was clearly underpowered for this trait. Moreover, there were no significant imprinting variances for the net BW gain, which agreed with the findings of Engellandt and Tier (2002) in German Gelbvieh. However, comparisons with different breeds should be treated with caution because there is a great emphasis on milk performance in Brown Swiss, whereas German Simmental and German Gelbvieh are dual-purpose breeds, with some focus on the beef performance.

The relative imprinting variance determined by Neugebauer *et al*. (2010b) for the conformation class was similar to our results, but their estimated relative imprinting variance for the fat score (24.77%) was more than double the proportion estimated for the fat score in our study. In addition to differences in the breed backgrounds, this may be explained by the fact that different recording techniques were used. Thus, instead of using five visually observed scores, Neugebauer *et al*. (2010b) employed 15 automatically video-recorded categories, which probably captured the actual degree of phenotypic variation with much greater precision.

### Allelic contributions to the imprinting variance

The parental contributions of gametes to the imprinting variance can be calculated as 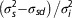 for the paternal contribution and 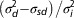 for the maternal contribution. For the net BW gain, the relative contribution of maternal alleles to the imprinting variance was almost exactly 100% (Table 2).

For the carcass quality traits, the maternal gamete contributed 87.7% to the imprinting variance in the fat score and 92.7% to the imprinting variance in the conformation class (Table 2). The standard errors of these contributions were larger (22.2% and 28.3%) than the respective paternal contributions of 12.3% and 7.3%. However, the results obtained by GLMM were different, where the covariance between paternal and maternal effects was larger than the paternal variance, thereby resulting in negative paternal contributions of −35.7% for the fat score and −37.9% for the carcass conformation. Both values exceeded their standard errors (20.7% and 20.0%, respectively) in magnitude by about one-third or more. Thus, the maternal contributions for both traits were almost exactly four-thirds.

Our findings differed from those obtained by Neugebauer *et al*. (2010b), who attributed most of the imprinting variance in the fat score and conformation class to paternal gametes in dual-purpose German Simmental. However, our results obtained in Brown Swiss agreed with an analysis based on ultrasonic measures of body composition in Australian beef cattle (Tier and Meyer, 2012). In principle, maternal genetic effects can lead to overestimates of the imprinting variance (Hager *et al*., 2008), and thus the estimated maternal contributions. On the other hand, unaccounted paternally inherited effects may lead to biased estimates for variance components in models with maternal genetic and direct effects (Varona *et al*., 2015). We cannot rule out the existence of maternal genetic effects in the Brown Swiss data set by own investigations. They are, however, generally considered unimportant and they are not included in the models used for routine genetic evaluations for that breed, mainly on the practical grounds that the separation of calves from their dams shortly after birth is a common practice and they are raised with a formula diet. It should also be noted that Tier and Meyer (2012) attributed the occurrence of negative contributions (the covariance exceeds one of both variances) to the effects of partially imprinted loci because fully imprinted loci only contribute to the variances.

### Heritability

To estimate the heritability (*h* ^2^) for all of the given slaughter traits, we first used linear animal models. The estimated values of *h* ^2^ obtained from animal models are summarised in Table 1. The results obtained from the LMM were 0.52 for the killing out percentage, 0.26 for net BW gain, 0.22 for fat score and 0.15 for conformation class. GLMM obviously captured a larger proportion of the genetic variability with estimates of 0.46 for the fat score and 0.43 for conformation class. The standard errors were not >0.02, with the exception of the killing out percentage with 0.08. The resulting estimates were quite similar (Table 2) when imprinting was part of the model, with a small increase of about 1% compared with the results obtained by the LMM. The standard errors of the heritabilities also remained about the same (Table 2).

To the best of our knowledge, the genetic parameters of beef traits have not been reported previously for Brown Swiss cattle. In German Simmental, Neugebauer *et al*. (2010b) estimated an *h* ^2^ value of 0.22 for the killing out percentage using the same imprinting model. A considerably higher value of 0.50 was found for the dressing percentage in German Gelbvieh fattening bulls (Engellandt *et al*., 1999b), which are raised under comparable production circumstances. The latter estimate agrees well with our result of slightly >50% for Brown Swiss. Neugebauer *et al*. (2010b) estimated *h* ^2^ values of 0.28 for the net BW gain and 0.25 for the fat score in German Simmental. These results agree almost perfectly with our results. However, for the conformation class, their estimate of 0.31 was double our estimate of about 0.15. As mentioned earlier, Neugebauer *et al*. (2010b) automatically video recorded the carcass quality traits according to a scale with 15 different categories, whereas our data comprised a coarse subjective categorisation with only five categories. Despite this difference, the GLMM picked up a high *h* ^2^ value of 0.43, which was similar to the estimate obtained by Neugebauer *et al*. (2010b). In contrast, lower *h* ^2^ values of 0.22 and 0.12 were obtained for the conformation class and fat score in a Bayesian analysis of German Simmental data (Reinsch *et al*., 1999), where both traits were treated as dichotomous binary traits.

### Generalised linear mixed model *v.* linear mixed model

**Figure 3:**
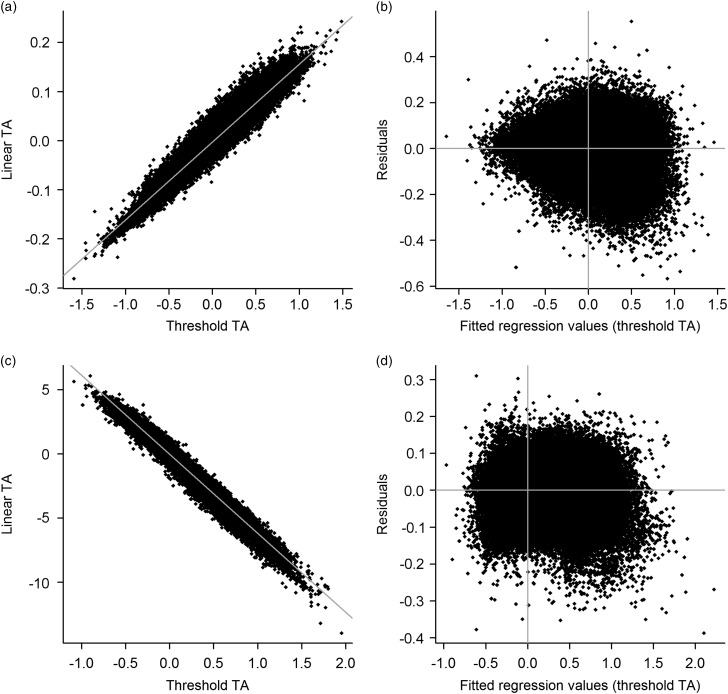
Correlations between transmitting abilities (TA, left side) estimated using a linear (linear TA) and a generalised linear mixed model (threshold TA). The threshold TA was fitted using the linear TAs as independent variables with respect to their residuals (right side) for the fat score (a, b) and conformation class (c, d).

We determined high correlations between the POE predicted using the *equivalent model* as LMM and using the *equivalent model* as GLMM, with values of 0.95 for the fat score and −0.90 for the conformation class (the negative sign is due to the reversed order of categories in the GLMM). High correlations were also obtained for the reliability of the POE, with values of 0.98 and 0.97. The clear linear relationships between the TA from the GLMM and the TA from the LMM can be seen in Figure 3 for the fat score (a) and conformation class (b). The residual variation in the TA obtained from the GLMM regressed on the TA from LMM was fairly constant over the entire range for the conformation class (Figure 3d), whereas the variation increased slightly more for the fat score for larger TA (Figure 3b). Overall, these comparisons demonstrate that both models accounted for a large proportion of the same type of variation, although there was no full agreement in terms of the respective estimates of genetic effects.

The LMM yields estimates that can be interpreted directly in terms of monetary differences but this is not the case with the GLMM. Kempster *et al*. (1982) reported that the conformation class only explained ~30% of the variation in meat content. Therefore, from a biological perspective, the nature of the underlying continuity (Falconer, 1960) is not clear for the conformation class. In contrast, Drennan *et al*. (2008) reported a high positive correlation (*r*=0.83) between the carcass fat score and carcass fat proportion in bulls, and thus it is plausible that the underlying continuous variable for the fat class is generally identical to the carcass fatness. However, the approximate monetary value of a 1 unit change in the TA on the underlying scale for the carcass conformation can be derived by assessing the associated changes in the average frequencies for all categories and in the average value of a carcass.

## Conclusion

In this study, we developed a new model that facilitates the direct estimation of imprinting effects and PEVs for a large number of animals using existing software. Furthermore, we determined significant imprinting variances for three of four beef traits analysed in Brown Swiss fattening bulls using LMM and GLMM. The imprinting variances accounted for ~10% of the total genetic variance, where the maternal gametes provided the largest contributions. These findings highlight the importance of POE and support the need to incorporate them into selection decisions.

## Supplementary material

For supplementary material accompanying this paper visit http://dx.doi.org/10.1017/S1751731116002391. click here to view supplementary material
