# Supplementary material for: A new model for parent-of-origin effect analyses applied to Brown Swiss cattle slaughterhouse data
Source: Animal. 2016 Dec 6;11(7):1096–106. doi: 10.1017/S1751731116002391 (PMC5488768; doi:10.1017/S1751731116002391)
Supplement: Supplementary file 1 [file S1751731116002391sup001.docx]

A new model for parent-of-origin effect analyses applied to Brown Swiss cattle slaughterhouse data

I. Blunk, M. Mayer, H. Hamann and N. Reinsch

**Supplementary material**

**Proof of model equivalence**

Model equivalence in Henderson’s sense (1985) requires the expectation $E\left(y\right)$ and variance $Var\left(y\right)$ of the observation vector to be identical under the specifications of both models. Both models do not differ in their fixed effects parts and all random variables have zero expectations. Therefore, $E\left(y\right)$ is obviously equal for the *imprinting model* and the *equivalent model* with:

$E\left(y\right)=X\beta$.

The variance of the observations $Var\left(y\right)$ in the *imprinting model* can be written as:

$\left[\begin{matrix} Z_{s} & Z_{d} \end{matrix}\right]\left[1/2A\otimes\left(\begin{matrix} \sigma_{s}^{2} & \sigma_{sd} \\ \sigma_{sd} & \sigma_{d}^{2} \end{matrix}\right)\right]\left[\begin{matrix} Z_{s}^{'} \\ Z_{d}^{'} \end{matrix}\right]+W\sigma_{R}^{2}$.

The second part of the latter sum reflects the residual variation and is identical in both models, as already explained. The first part is associated with the numerator relationship matrix $A$ and depicts the covariance of observations due to the resemblance of relatives. After multiplication this covariance becomes:

| $Z_{s}1/2A\sigma_{s}^{2}Z_{s}^{'}+Z_{d}1/2A\sigma_{sd}Z_{s}^{'}+Z_{s}1/2A\sigma_{sd}Z_{d}^{'}+Z_{d}1/2A\sigma_{d}^{2}Z_{d}^{'}$. | (1) |
| --- | --- |

The variance of the observations $Var\left(y\right)$ in the *equivalent model* can be expressed in terms of the incidence matrices and variance components from the *imprinting model*. In particular, the incidence matrix for the TA of sires as sires in the *equivalent model* is the sum of $Z_{s}$ and $Z_{d}$ in the *imprinting model*, and the incidence matrix $Z_{i}$ for imprinting effects in the *equivalent model* equals $Z_{d}$ from the *imprinting model*, as explained before. Therefore, $Var\left(y\right)$ in the *equivalent model* becomes:

$\left[\begin{matrix} \left(Z_{s}+Z_{d}\right) & Z_{d} \end{matrix}\right]\left[1/2A\otimes\left(\begin{matrix} \sigma_{s}^{2} & \sigma_{sd}-\sigma_{s}^{2} \\ \sigma_{sd}-\sigma_{s}^{2} & \sigma_{s}^{2}+\sigma_{d}^{2}-2\sigma_{sd} \end{matrix}\right)\right]\left[\begin{matrix} \left(Z_{s}+Z_{d}\right)^{'} \\ Z_{d}^{'} \end{matrix}\right]+W\sigma_{R}^{2}$.

Again we consider only the first part of this sum, which is associated with $A$, in detail. Multiplication gives:

$$\begin{matrix} \left(Z_{s}+Z_{d}\right)1/2A\sigma_{s}^{2}\left(Z_{s}+Z_{d}\right)^{'}+\left(Z_{s}+Z_{d}\right)1/2A\left(\sigma_{sd}-\sigma_{s}^{2}\right)Z_{d}^{'}+Z_{d}1/2A\left(\sigma_{sd}-\sigma_{s}^{2}\right)\left(Z_{s}+Z_{d}\right)^{'} \\ +Z_{d}1/2A\left(\sigma_{s}^{2}+\sigma_{d}^{2}-2\sigma_{sd}\right)Z_{d}^{'} \\ \\ =Z_{s}1/2A\sigma_{s}^{2}Z_{s}^{'}+Z_{s}1/2A\sigma_{s}^{2}Z_{d}^{'}+Z_{d}1/2A\sigma_{s}^{2}Z_{s}^{'}+Z_{d}1/2A\sigma_{s}^{2}Z_{d}^{'} \\ +Z_{s}1/2A\sigma_{sd}Z_{d}^{'}-Z_{s}1/2A\sigma_{s}^{2}Z_{d}^{'}+Z_{d}1/2A\sigma_{sd}Z_{d}^{'}-Z_{d}1/2A\sigma_{s}^{2}Z_{d}^{'} \\ +Z_{d}1/2A\sigma_{sd}Z_{s}^{'}-Z_{d}1/2A\sigma_{s}^{2}Z_{s}^{'}+Z_{d}1/2A\sigma_{sd}Z_{d}^{'}-Z_{d}1/2A\sigma_{s}^{2}Z_{d}^{'} \\ +Z_{d}1/2A\sigma_{s}^{2}Z_{d}^{'}+Z_{d}1/2A\sigma_{d}^{2}Z_{d}^{'}-2Z_{d}1/2A\sigma_{sd}Z_{d}^{'} \\ \\ =Z_{s}1/2A\sigma_{s}^{2}Z_{s}^{'}+Z_{d}1/2A\sigma_{sd}Z_{s}^{'}+Z_{s}1/2A\sigma_{sd}Z_{d}^{'}+Z_{d}1/2A\sigma_{d}^{2}Z_{d}^{'}. q.e.d. \end{matrix}$$

The latter is identical to term 1. In conclusion both conditions from Henderson (1985) are fulfilled and model equivalence has been formally proofed.

**References**

Henderson CR 1985. Equivalent linear models to reduce computations. Journal of Dairy Science 68, 2267–2277.
